# Passive immunotherapy of extended peritonitis as abdominal sepsis prevention

**DOI:** 10.1186/cc12906

**Published:** 2013-11-05

**Authors:** Olexandr Butyrsky, Viktor Starosek

**Affiliations:** 1 Department of Surgical Diseases, Crimean State Medical University, Simferopol, Ukraine

## Background

The outcome of extended peritonitis is determined by many factors including antimicrobial defense. Microbial invasion, surgery, and intensive therapy cause secondary immunity deficiency associated with septic complication incidence and post-surgery lethality. The great importance in initialization and supporting these processes belongs to *Escherichia coli *endotoxin that participates in digestive tract immunity and general immunoresistance.

## Materials and methods

Thirty-two patients ages 15 to 86 (male:female = 24:8) treated for extended peritonitis were investigated. Blood was sampled after admission and in 5 days to determine anti-lipopolysaccharide antibodies of different classes (anti-LPS-IgA, anti-LPS-IgG, anti-LPS-IgM, respectively) by hard-phase immunoenzyme analysis. The control group included 10 healthy donors (opt.un.): anti-LPS-IgA - 0.348 ± 0.053, anti-LPS-IgM - 0.162 ± 0.01, anti-LPS-IgG - 0.333 ± 0.051.

## Results

Patients with high levels of anti-endotoxin immunity were 15.6% (*n *= 5) (Table 1); after surgery they had rapid recovery, normalization of peristalsis and laboratory parameters by the 5th day. Patients of low immunity level were 84.4% (*n *= 27); they had a long complicated recovery period. In group I for standard treatment within 5 days one noticed evident shifts of all parameters that witnesses its sufficiency. In group II the parameters are not increased evidently, which testifies to necessity of additional immunocorrection. Low immunity level patients were introduced to 3 ml sandoglobulin H on the 5th day after surgery that was associated with a sharp increase of anti-LPS antibody titer (Table 2). Growth of anti-LPS antibody titer was associated with positive dynamics of the post-surgery period.

**Table 1:** Indexes of anti-endotoxin immunity in extended peritonitis

	**Before treatment, opt.un**.	**5th day of treatment, opt.un**.
High immunity level patients (group I, *n *= 5)		
Anti-LPS-IgA	0.276 ± 0.004 (p_1 _> 0.05)	0.452 ± 0.02 (p_2 _< 0.001)
Anti-LPS-IgM	0.210 ± 0.03 (p_1 _< 0.01)	0.286 ± 0.04 (p_2 _< 0.05)
Anti-LPS-IgG	0.121 ± 0.01 (p_1 _< 0.01)	0.184 ± 0.02 (p_2 _< 0.01)
Low immunity level patients (group II, *n *= 27)		
Anti-LPS-IgA	0.084 ± 0.007 (p_1 _< 0.001, p_3 _< 0.001)	0.154 ± 0.015 (p_2 _< 0.01)
Anti-LPS-IgM	0.202 ± 0.02 (p_1 _< 0.05, p_3 _> 0.05)	0.213 ± 0.01 (p_2 _> 0.05)
Anti-LPS-IgG	0.069 ± 0.008 (p_1 _< 0.01, p_3 _< 0.001)	0.083 ± 0.007 (p_2 _> 0.05)

p_1_, evidence between donors and day of admission; p_2_, evidence between day of admission and the 5th day; p_3_, evidence between patients of different immunity level.

**Table 2:** (abstract P5). Dynamics of anti-LPS-antibodies in low immunity level patients with peritonitis after sandoglobulin H injection

	**Before injection, opt.un**.	**After injection, opt.un**.
Anti-LPS-IgA	0.154 ± 0.015	0.342 ± 0.02*
Anti-LPS-IgM	0.213 ± 0.01	0.284 ± 0.02*
Anti-LPS-IgG	0.083 ± 0.007	0.186 ± 0.04*

*P < 0.001.

## Conclusions

The majority of peritonitis patients have decreased competent anti-LPS antibodies, which determines the severity of the post-surgery period. Low immunity level patients need passive nonspecific immunotherapy that stimulates protective functions, blocks mechanisms of inflammation progress, and prevents abdominal sepsis.

